# Improvement of dexamethasone sensitivity by chelation of intracellular Ca^2+^ in pediatric acute lymphoblastic leukemia cells through the prosurvival kinase ERK1/2 deactivation

**DOI:** 10.18632/oncotarget.16039

**Published:** 2017-03-09

**Authors:** Souleymane Abdoul-Azize, Isabelle Dubus, Jean-Pierre Vannier

**Affiliations:** ^1^ Micro-Environnement et Renouvellement Cellulaire Intégré, MERCI UPRES EA 3829, Faculté de Médecine et Pharmacie, Université de Rouen, 76183 Rouen Cedex, France; ^2^ Service Immuno-Hémato-Oncologie Pédiatrique, CHU Charles Nicolle, 76031 ROUEN Cedex, France; ^3^ Current address: Unité Inserm U1234/Université de Rouen/IRIB, Rouen, France

**Keywords:** acute lymphoblastic leukemia, dexamethasone, Ca^2+^ signaling, ERK1/2 pathway, apoptosis

## Abstract

Previous studies have demonstrated that glucocorticoid hormones, including dexamethasone, induced alterations in intracellular calcium homeostasis in acute lymphoblastic leukemia (ALL) cells. However, the mechanism by which intracellular calcium homeostasis participates in dexamethasone sensitivity and resistance on ALL cells remains elusive. Here, we found that treatment of cells with dexamethasone resulted in increased intracellular calcium concentrations through store-operated calcium entry stimulation, which was curtailed by store-operated calcium channel blockers. We show that BAPTA-AM, an intracellular Ca^2+^ chelator, synergistically enhances dexamethasone lethality in two human ALL cell lines and in three primary specimens. This effect correlated with the inhibition of the prosurvival kinase ERK1/2 signaling pathway. Chelating intracellular calcium with Bapta-AM or inhibiting ERK1/2 with PD98059 significantly potentiated dexamethasone-induced mitochondrial membrane potential collapse, reactive oxygen species production, cytochrome c release, caspase-3 activity, and cell death. Moreover, we show that thapsigargin elevates intracellular free calcium ion level, and activates ERK1/2 signaling, resulting in the inhibition of dexamethasone-induced ALL cells apoptosis. Together, these results indicate that calcium-related ERK1/2 signaling pathway contributes to protect cells from dexamethasone sensitivity by limiting mitochondrial apoptotic pathway. This report provides a novel resistance pathway underlying the regulatory effect of dexamethasone on ALL cells.

## INTRODUCTION

Glucocorticoids are the most common compounds used in the treatment of lymphoid malignancies, including acute lymphoblastic leukemia (ALL), because of their abilities to induce cell death [1]. However, up to ∼20% patients with leukemia relapse and become resistant to glucocorticoids [2]. Glucocorticoid-induced apoptosis is generally via a genomic mechanism and mediated by the activation of glucocorticoid receptor (GR), which is, in the cytosol, sequestered and bound to heat shock proteins in its inactive state [3]. GR is expressed throughout all cells of the body [4], including B cells [2]. When glucocorticoid binds to the GR, the complex is translocated to the nucleus to activate or inhibit target genes transcription [5].

Calcium ions are key regulators of several cellular mechanisms, including cell growth and death [6]. It has been reported that glucocorticoids (dexamethasone or corticosterone) induced a decrease in [Ca^2+^]i in neurons and astrocytes [7], and in T lymphocytes [8]. These observations were opposite to many reports indicating that glucocorticoid increases Ca^2+^ signaling in murine [9] and human [10] lymphoma cell lines. Thereby, how the Ca^2+^ signaling participates in glucocorticoids-induced cell death is not entirely clear. Previous studies from our group demonstrated that TRPC3 Ca^2+^ channel inhibitor (Pyr3) enhances dexamethasone sensitivity and apoptosis through the distraction of dexamethasone-mediated Ca^2+^ signaling in ALL cells and primary blasts [11]. Because we demonstrated that depletion of intracellular calcium increased by dexamethasone, enhanced dexamethasone-mediated apoptosis of ALL cells [11], we here studied the mechanism by which intracellular calcium signal participates in dexamethasone sensitivity and resistance in ALL cells.

The mitogen-activated protein kinase (MAPK) signaling pathway regulates many cellular processes including proliferation, differentiation, inflammation, cell stress response, cell division, metabolism, motility and apoptosis [12]. Increasing evidence reveals the implication of mitogen-activated protein kinases (MAPKs) pathway in the regulation of glucocorticoids sensitivity in ALL cells [1, 2], but the mechanism by which MAPKs activate or inhibit glucocorticoids sensitivity and resistance remains unknown. In several cell types, as well as cancer cells, ERK1/2 signaling pathway is a significant downstream substrate for Ca^2+^ ions, thus, increases in [Ca^2+^]i phosphorylated ERK1/2 proteins [13, 14]. Therefore, in the present work, we investigated whether dexamethasone induces apoptosis is attenuated by Ca^2+^-dependent activation of MAPK/ERK pathway. We report here, that calcium-related ERK1/2 signaling pathway contributes to protect leukemic cells from dexamethasone sensitivity, describing a new mechanism of resistance pathway underlying the regulatory effect of dexamethasone on ALL cells.

## RESULTS

### Bapta-AM increases sensitivity to dexamethasone in ALL cell lines

**Figure 1:**
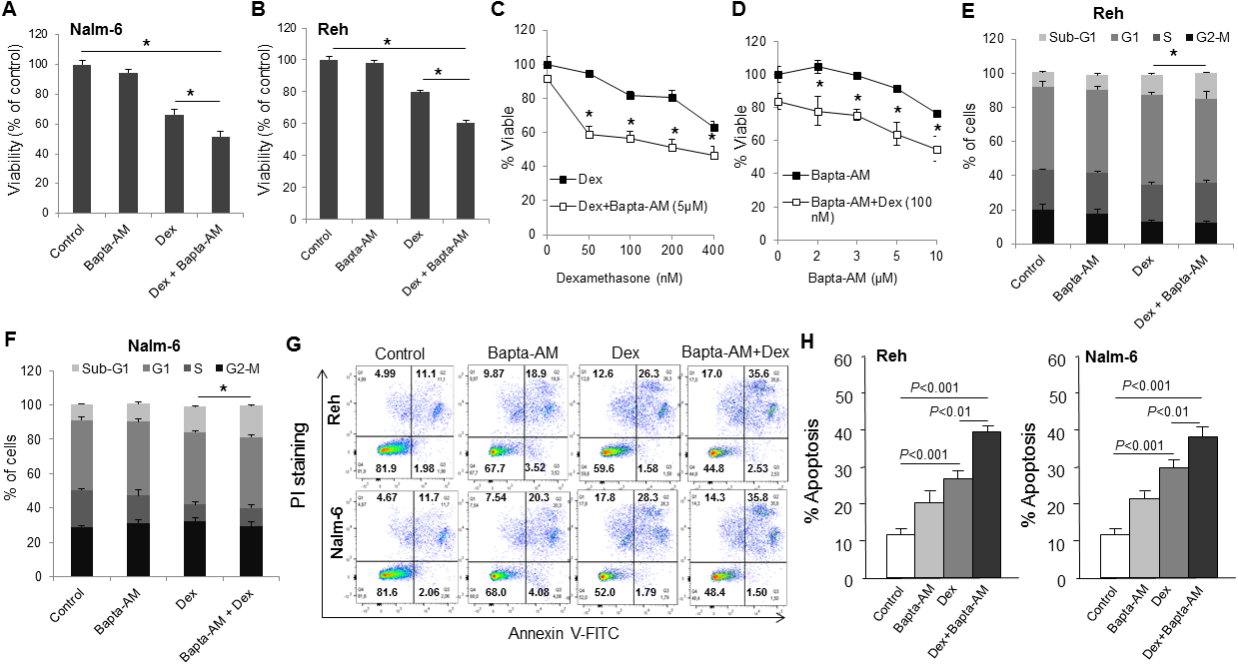
Bapta-AM markedly enhances dexamethasone-induced cell death, cell cycle disruption and apoptosis in ALL cell lines Nalm-6 **(A)** and Reh **(B)** cells were treated with Bapta-AM (5 μM) and dexamethasone (Dex, 100 nM) alone or in combination for 48 h. Cell death was detected by MTT metabolic colorimetric assay. Data are representative of triplicate experiments. **(C)** ALL Reh cells were treated for 48 h with increasing concentrations of dexamethasone (Dex) alone or in association with Bapta-AM (5 μM). **(D)** ALL Reh cells were treated for 48 h with indicated concentration of Bapta-AM alone or in association with dexamethasone (Dex, 100 nM). The values of control cells were considered as 100%. Data represent the means ±S.E.M. (n=3). *P<0.05 *vs* Dex or Bapta-AM alone treatment. Cell cycle distribution **(E, F)** and apoptosis **(G, H)** were determined respectively by PI staining and Annexin V/FITC-PI staining followed by FACS analysis. *P<0.05 *vs* dexamethasone alone treatment **(E, F)**. **(H)** The percentage of apoptotic cells was calculated by the percentage of annexin V-FITC positive and annexin V-FITC/PI-positive population. Combination index (CI) value < 1 (0.58 in Nalm-6 and 0.45 in Reh cells) indicates that the drugs are significantly synergistic. Data represent the mean ± S.E.M. (n=3).

Our recent studies have shown that TRPC3 Ca^2+^ channel inhibitor (Pyr3) enhances dexamethasone sensitivity in ALL cells by reducing Ca^2+^ signaling [11]. To investigate the role of cytosolic Ca^2+^ in dexamethasone-induced leukemic cells apoptosis, first of all, we treated ALL cells with or without dexamethasone for 48 h following pre-incubation with or without 1,2-bis (2-aminophenoxy) ethane-N,N,N,N-tetraacetic acid (Bapta-AM), an intracellular Ca^2+^ chelator. We found that ALL cell lines viability was not significantly modified by a 48-hours treatment with Bapta-AM (5 μM) alone, while treatment with dexamethasone (100 nM) alone decreased cell viability in both Nalm-6 (Figure 1A) and Reh (Figure 1B) cell lines. However, association of dexamethasone with Bapta-AM markedly decreased cell viability when compared to dexamethasone alone (Figure 1A, 1B). The effect of dexamethasone or Bapta-AM alone on cell death was dose-dependent and co-treatment potentiated this effect. Concentration of dexamethasone as low as 50 nM significantly decreased cell viability in association with 5 μM of Bapta-AM (Figure 1C). The dose-response graph of Bapta-AM indicated that concentrations as low as 5 μM Bapta-AM significantly increased the lethality of 100 nM dexamethasone in ALL cell lines (Figure 1D). Furthermore, dexamethasone in association with Bapta-AM induced significant amount of sub-G1 apoptotic cell population compared with dexamethasone alone treatment (Figure 1E, 1F). To confirm the role of Bapta-AM in dexamethasone-induced ALL cell lines apoptosis, we analyzed the apoptotic status in these cells using Annexin V-FITC/PI staining. The apoptosis rate was slightly increased with Bapta-AM or dexamethasone alone treatment, but significantly increased by co-treatment in both ALL cell lines when compared to control or dexamethasone alone (Figure 1G, 1H), confirming the results obtained by the MTT assay. We next examined whether the effect on cell death induced by Bapta-AM with dexamethasone is synergistic or additive. Isobologram analysis [15] was used to analyze effects of drug combinations. Combination index (CI) value <1, =1 or >1 indicates that the drugs are synergistic, additive or antagonistic, respectively. The combination of 5 μM Bapta-AM with 100 nM of dexamethasone produced a significant synergistic effect in Nalm-6 and Reh cells with a combination index ranging from 0.58 to 0.45, respectively (Figure 1G).

### Bapta-AM increases dexamethasone-induced apoptosis via regulating mitochondrial functions in ALL cell lines

**Figure 2:**
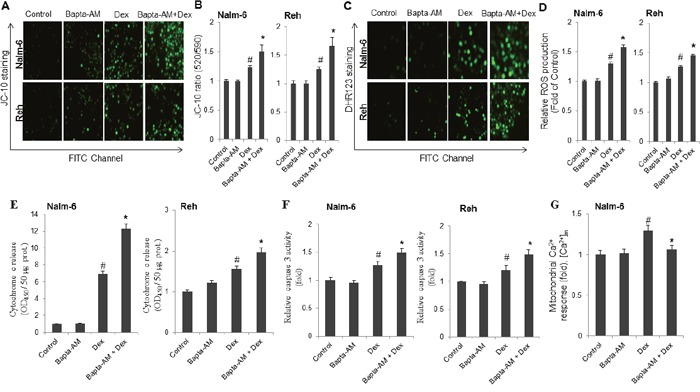
Co-treatment with dexamethasone and Bapta-AM markedly increases mitochondrial membrane potential depolarization, reactive oxygen species production, cytochrome c release and caspase 3 activity in ALL cells Cells were treated with Bapta-AM (5 μM) and dexamethasone (Dex, 100 nM) alone or in combination for 24 h. Images acquired with Zeiss Axiovert 200M fluorescence microscope after JC-10 **(A)** and DHR 123 **(C)** staining using FITC channel. The fluorescence intensity for both mitochondrial membrane potential changes **(B)** and intracellular reactive oxygen species generation **(D)** was measured with SAFAS Xenius XC Spectrofluorometer. The bar graphs of mean fluorescence intensity representing cytochrome c release **(E)** caspase-3 activity **(F)** and mitochondrial calcium **(G)**. Data represent the mean ± S.E.M. (n=3). *P<0.05 *vs* dexamethasone alone treatment; ^#^P<0.05 *vs* control.

Because of the fundamental role of mitochondria in cell apoptosis, we next determined whether the effect of Bapta-AM on dexamethasone-induced ALL cells apoptosis was mediated through modulating mitochondrial functions. To this end, ALL cells were pretreated with or without Bapta-AM (5 μM) for 30 min and then exposed to dexamethasone (100 nM) for 24 h. The dissipation of mitochondrial membrane potential (Δψ_m_), an early event for cell apoptosis, was detected by JC-10, a lipophilic cationic dye. As shown in Figure 2A, a green fluorescence represents depolarized mitochondria in ALL cells. In agreement with the apoptosis results, dexamethasone-induced Δψ_m_ collapse (Figure 2A, 2B) was significantly enhanced by the intracellular Ca^2+^ chelator Bapta-AM. As the loss of mitochondrial membrane potential is known to trigger reactive oxygen species (ROS) production [16], the possible implication of ROS in ALL cells apoptosis induced by dexamethasone in the presence of Bapta-AM was investigated. By using cell permeable dihydrorhodamine 123 (DHR123), a green fluorescence probe, we found that Bapta-AM enhanced the ability of dexamethasone to induce ROS production (Figure 2C, 2D). A consequence of ROS production and Δψ_m_ collapse is the initiation of mitochondria-mediated cell apoptosis cascade in which cytochrome c release and caspase-3 activity play a critical role [17]. We next determined whether the influence of Bapta-AM on dexamethasone-induced apoptosis is associated with the release of cytochrome c and the activity of caspase-3. As shown in Figure 2, both cytochrome c release (Figure 2E) and caspase-3 activity (Figure 2F) induced by dexamethasone were markedly potentiated by Bapta-AM. These data, together with the results obtained above, suggest that the intracellular Ca^2+^ contributes to attenuate dexamethasone-induced apoptosis in ALL cells by limiting Δψ_m_ collapse, ROS production, and cytochrome c release from mitochondria followed by caspase-3 activity. Furthermore, the potentiating effect of dexamethasone-mediated apoptosis with Bapta-AM may not depend on mitochondrial calcium release in ALL cells, indeed, as shown in Figure 2G, measurement of mitochondrial Ca^2+^ indicated that the intracellular Ca^2+^ chelator notably abolished dexamethasone-mediated mitochondrial Ca^2+^ release.

### Dexamethasone induces cytosolic calcium release and SOCE and co-treatment with dexamethasone and SOC inhibitors markedly enhances ALL cells death

**Figure 3:**
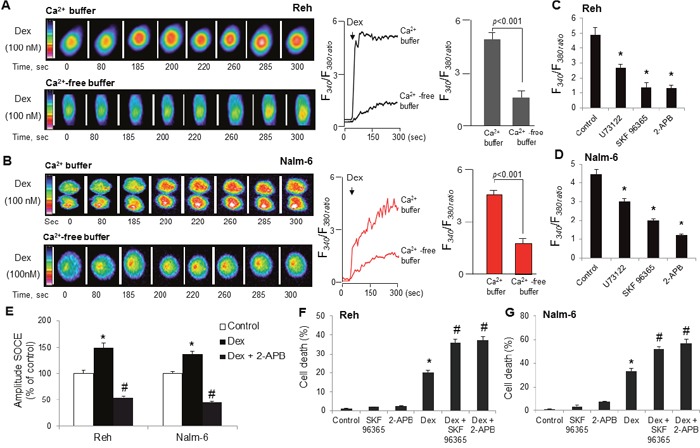
Dexamethasone stimulates intracellular Ca2+ release and SOCE and co-treatment with dexamethasone and SOC inhibitors markedly enhances ALL cells death Cells were loaded with Fura-2/AM, and the changes in intracellular Ca^2+^, [Ca^2+^]i, (F340/F380) were monitored. **(A, B)** The colored time-lapse images and the graphical representation show the changes in [Ca^2+^]i evoked by dexamethasone (Dex), in Reh and Nalm-6 cell lines, respectively, in Ca^2+^-containing and in Ca^2+^-free buffer. **(C, D)** ALL cells before exposure to 100 nM dexamethasone (Dex) in Ca^2+^ buffer were preincubated (20 minutes) with U73122 (10 μM), SKF96365 (10 μM) or 2-APB (10 μM). Data are mean ± SEM (n = 5). **(E)** Effect of dexamethasone (Dex) on SOCE activation in ALL cells. ALL cells ER calcium stores were depleted with thapsigargin (TG, 1 μM) in calcium-free suspension medium in the presence or absence of 2-APB (10 μM), cells were then treated without or with 100 nM dexamethasone, followed by addition of 1.8 mM CaCl_2_. Data are mean ± SEM (n = 3). Reh **(F)** and Nalm-6 **(G)** cells were treated with SOC inhibitors (SKF 96365, 5 μM and 2-APB, 5 μM) and dexamethasone (100 nM) alone or in combination for 48 h. Cell death was detected by MTT metabolic colorimetric assay. Data are representative of triplicate experiments. *p <0.001 *vs*. control; #p <0.001 *vs*. Dex.

We next sought to examine the effect of dexamethasone on Ca^2+^ signaling in ALL cell lines. As shown in Figure 3A and 3B, addition of dexamethasone evoked an increase in intracellular free Ca^2+^ concentrations ([Ca^2+^]i) in both ALL cell lines, and dexamethasone-induced increases in [Ca^2+^]i were significantly higher in Ca^2+^-containing as compared with Ca^2+^-free buffer (Figure 3A, 3B), suggesting that dexamethasone significantly raised the peak of the Ca^2+^ elevation resulting from extracellular Ca^2+^ influx. To elucidate whether dexamethasone-mediated intracellular calcium elevation is contributed, as per capacitative model, by the opening of store-operated Ca^2+^ (SOC) channels, we conducted experiments in calcium buffer in the presence of SKF96365 and 2-aminoethoxydiphenyl borate (2-ABP), the SOC channel blockers [18, 19]. We observed that blockers of SOC channels significantly decreased dexamethasone-induced increases in [Ca^2+^]i in both ALL cell lines (Figure 3C, 3D). In addition, preincubation with U73122, an inhibitor of phospholipase C (PLC) significantly curtailed dexamethasone-induced increases in [Ca^2+^]i in these cells (Figure 3C, 3D). To further examine whether store operated Ca^2+^ entry (SOCE) is activated following dexamethasone stimulation in ALL cells, SOCE was assessed according to the commonly used protocol [20]. Thus, endoplasmic reticulum (ER) Ca^2+^ store is first completely depleted in Ca^2+^ free medium by application of 1 μM thapsigargin (TG, a noncompetitive inhibitor of the sarco/ER Ca^2+^-ATPase) and SOCE is then evaluated by addition of 1.8 mM CaCl_2_ in the extracellular medium. As illustrated in Figure 3E, dexamethasone significantly potentiated the peak of TG-induced SOCE. Moreover, when ALL cells were exposed to 2-APB (SOC blocker), the dexamethasone-induced increase in SOCE was significantly blunted (Figure 3E). Taken together, these results suggest that the dexamethasone-stimulated increase in calcium entry is at least in part SOCE dependent. To validate the possibility that suppression of dexamethasone-activated SOCE can enhance the dexamethasone-mediated cell death, we employed two pharmacological inhibitors, SKF 96365 and 2-APB, of the Ca^2+^ SOC channels [18, 19]. We observed that ALL cell lines viability was not significantly modified by a 48 h treatment with 2-APB (5 μM) and SKF 96365 (5 μM) alone, while co-treatment with dexamethasone (100 nM) significantly decreased cell viability in both Reh (Figure 3F) and Nalm-6 (Figure 3G) cell lines at 48 hrs, compared to dexamethasone alone treatment.

### Bapta-AM potentiates the dexamethasone-induced inhibition of ERK1/2 signaling in ALL cells by chelating Ca^2+^ signaling

**Figure 4:**
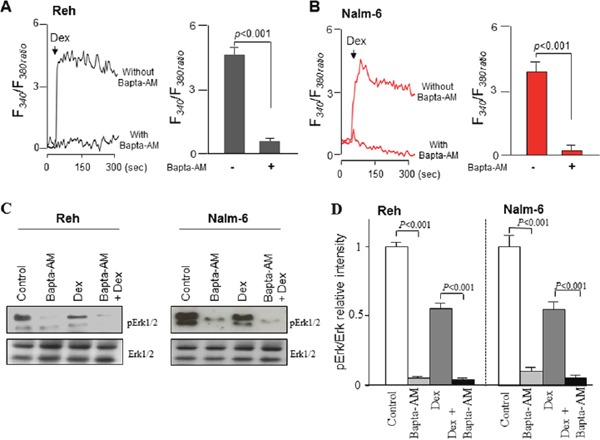
Bapta-AM potentiates dexamethasone-induced inhibition of ERK1/2 signaling by chelating Ca2+ signaling in ALL cells **(A, B)** Cells were exposed to 100 nM dexamethasone (Dex) with or without preincubated (20 minutes) with Bapta-AM (5 μM) in Ca^2+^ buffer and the changes in [Ca^2+^]**i** were monitored as Figure 5. **(C)** ALL cells were preincubated (20 min) with Bapta-AM (5 μM) in Ca^2+^ buffer and then exposed to 100 nM Dex for 5 min. Cell lysates were subjected to western blotting analysis with the indicated antibodies, along with quantification **(D)**. Data represent the mean ± SEM (n=3).

Since dexamethasone elicited strong [Ca^2+^]i signaling, we then examined the effect of Bapta-AM on dexamethasone-induced cytosolic Ca^2+^ elevation. To this end, ALL cells were pre-incubated with or without Bapta-AM (5 μM), an intracellular Ca^2+^ chelator, and then exposed or not to dexamethasone (100 nM). We observed that in the presence of Bapta-AM, dexamethasone failed to trigger cytosolic calcium elevation in both ALL cell lines (Figure 4A, 4B). In several cell types, as well as cancer cells, ERK1/2 signaling pathway is a significant downstream substrate for Ca^2+^ ions, thus, increases in [Ca^2+^]i phosphorylated ERK1/2 proteins [13]. We then examined whether Bapta-AM's Ca^2+^ chelation influenced the effect of dexamethasone on ERK1/2 phosphorylation. Interestingly, it was found that dexamethasone slightly decreased the phosphorylation of ERK1/2, whereas Bapta-AM completely suppressed ERK1/2 activation (Figure 6C, 6D), suggesting that Ca^2+^ is indeed a critical upstream factor that determined ERK1/2 phosphorylation. Concurrently, in comparison to dexamethasone alone treatment, Bapta-AM greatly enhanced dexamethasone-induced inhibition of ERK1/2 phosphorylation, which may be due to the inhibitory action of this Ca^2+^ chelator on dexamethasone-induced Ca^2+^ influx (Figure 4C, 4D). These results indicated that intracellular calcium attenuates dexamethasone-induced inhibition of ERK1/2. Suggesting that dexamethasone elevated cytosolic Ca^2+^ could trigger the activation of ERK1/2 signaling pathway.

### Pharmacological inhibition of ERK1/2 potentiates dexamethasone sensitivity and apoptosis in ALL cells

**Figure 5:**
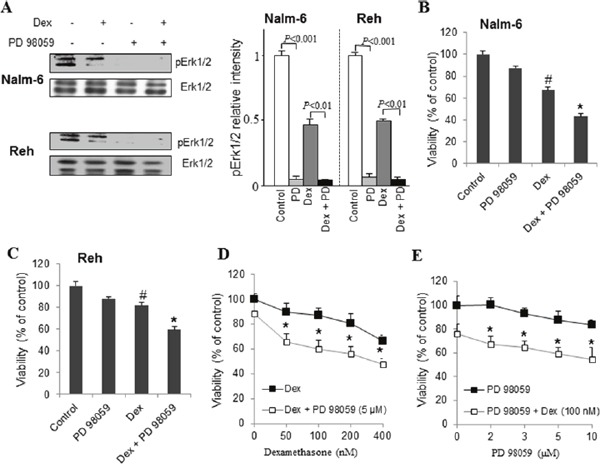
Pharmacological inhibition of ERK1/2 potentiates dexamethasone sensitivity in ALL cells ALL cells were preincubated (20 min) with PD98059 (5 μM) and then exposed to 100 nM dexamethasone (Dex) for 5 min. Cell lysates were subjected to western blotting analysis with the indicated antibodies, along with quantification **(A)**. Nalm-6 **(B)** and Reh **(C)** cells were treated with PD98059 (5 μM) and dexamethasone (100 nM) alone or in combination for 48 h. Cell death was detected by MTT metabolic colorimetric assay. Data are representative of triplicate experiments. **(D)** ALL Reh cells were treated for 48 h with increasing concentrations of dexamethasone (Dex) alone or in association with PD 98059 (5 μM). **(E)** ALL Reh cells were treated for 48 h with indicated concentration of PD98059 alone or in association with Dex (100 nM). The values of control cells were considered as 100%. Data represent the means ±S.E.M. (n=3). *P<0.05 *vs* PD 98059 or Dex alone treatment; #P<0.05 *vs* control.

In order to shed light on the involvement of ERK1/2 pathway in dexamethasone sensitivity and apoptosis, we used PD98059, a selective inhibitor of MEK. PD98059 has been shown to inhibit activation of MEK targets including ERK1/2 [1]. As shown in Figure 5A, pretreatment of ALL cells with PD98059 inhibited phosphorylation of ERK1/2 regardless of dexamethasone treatment. ALL cell lines viability was slightly modified by a 48-hours treatment with PD98059 (5 μM) alone, while treatment with dexamethasone (100 nM) alone decreased cell viability in both ALL cell lines (Figure 5B, 5C). However, association of dexamethasone and PD98059 markedly decreased ALL cells viability at the same time interval (Figure 5B, 5C). The effect of dexamethasone alone on cell death was dose-dependent and co-treatment with PD98059 potentiated this effect (Figure 5D). Concentration of dexamethasone as low as 50 nM significantly decreased cell viability in association with 5 μM of PD98059 (Figure 5D). The dose-response graph of PD98059 indicated that concentrations as low as 2 μM PD98059 significantly increased the lethality of 100 nM dexamethasone in ALL cell lines (Figure 5E).

**Figure 6:**
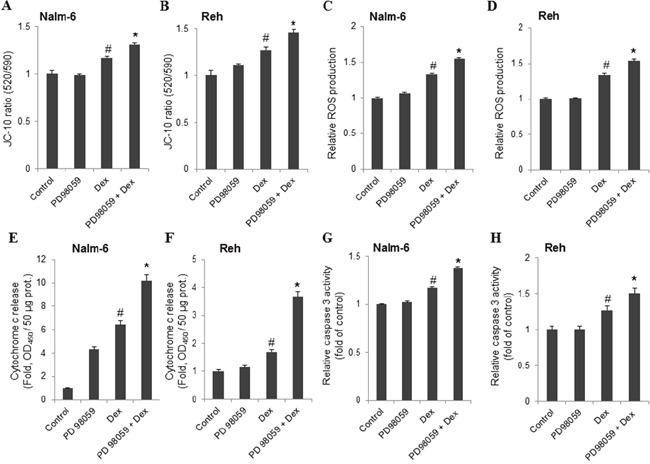
MEK inhibitor, PD98059, enhances dexamethasone-induced apoptosis in ALL cells ALL cells were treated with PD98059 (5 μM) and dexamethasone (Dex, 100 nM) alone or in combination for 24 h. The fluorescence intensity for mitochondrial membrane potential changes **(A, B)**, intracellular reactive oxygen species generation **(C, D)**, cytochrome c release **(E, F)** and caspase 3 activity **(G, H)** was measured with SAFAS Xenius XC Spectrofluorometer. Data represent the mean ± S.E.M. (n=3). *P<0.01 vs Dex alone treatment; #P<0.05 vs control.

We next examined whether the effects of MEK inhibitor on dexamethasone-induced ALL cells death were mediated through modulating mitochondrial functions. To this end, ALL cells were pretreated with or without PD98059 (5 μM) for 30 min and then exposed to dexamethasone (100 nM) for 24 h. As shown in Figure 6, and in agreement with the MTT results, dexamethasone-induced Δψm collapse (Figure 6A, 6B) and ROS production (Figure 6C, 6D) were significantly increased by PD98059 in ALL cells. A consequence of ROS production and Δψm collapse is the initiation of mitochondria-mediated cell apoptosis cascade in which cytochrome c release and caspase-3 activity play a critical role [17]. We next determined whether the influence of PD98059 on dexamethasone-induced ALL cells death is associated with the release of cytochrome c and the activity of caspase-3. As shown in Figure 6, both cytochrome c release (Figure 6E, 6F) and caspase-3 activity (Figure 6G, 6H) induced by dexamethasone were markedly potentiated by PD98059. These data indicate that ERK1/2 signaling pathway contributes to attenuate dexamethasone-induced apoptosis in ALL cells by limiting Δψm collapse, ROS production, and cytochrome c release from mitochondria followed by caspase-3 activity.

### Bapta-AM potentiates the dexamethasone-induced inhibition of ERK1/2 signaling and apoptosis in primary ALL cells

**Figure 7:**
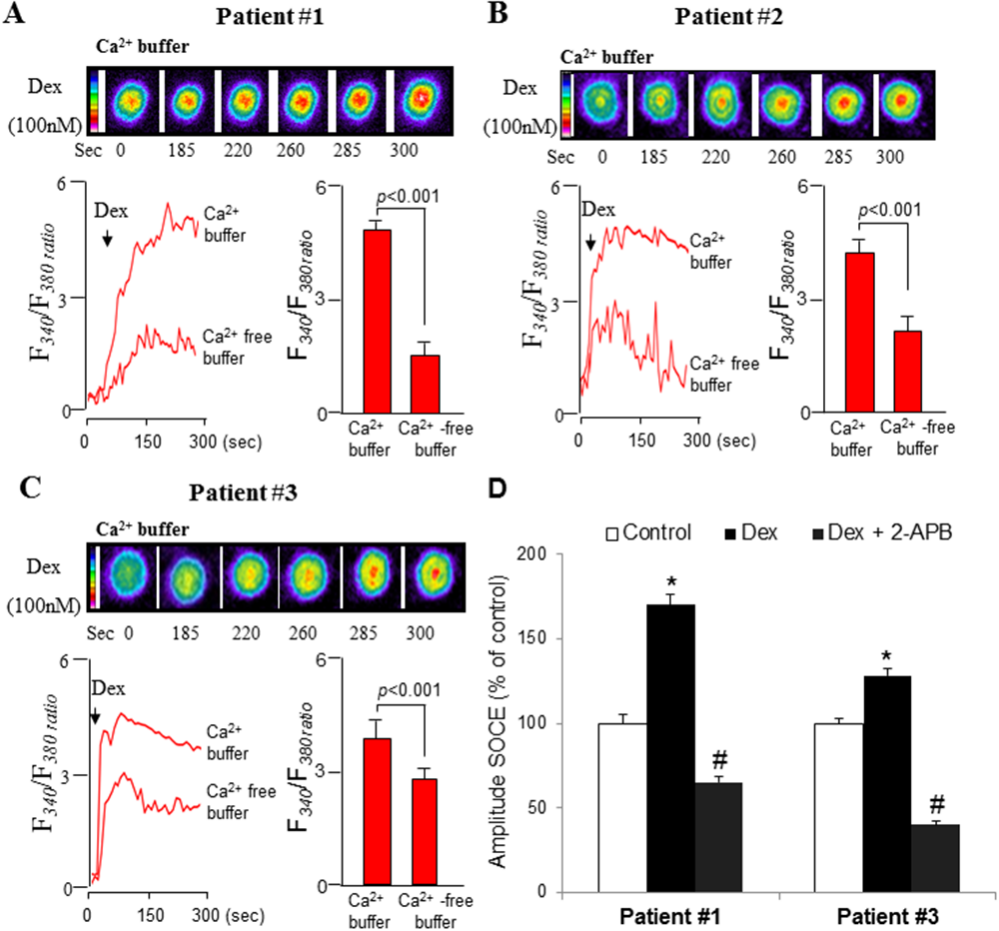
Dexamethasone stimulates intracellular Ca2+ release and SOCE in primary blasts from ALL patients Primary blasts were loaded with Fura-2/AM, and the changes in intracellular Ca^2+^, [Ca^2+^]i, (F_340_/F_380_) were monitored. The colored time-lapse images and the graphical representation show the changes in [Ca^2+^]i evoked by dexamethasone (Dex), respectively, in Ca^2+^-containing and in Ca^2+^-free buffer **(A, B, C)**. **(D)** Effect of Dex on SOCE activation. SOCE was monitored as Figure 3. (Data are mean ± SEM (n = 5). *p <0.001 vs. control; #p <0.001 vs. Dex.

**Figure 8:**
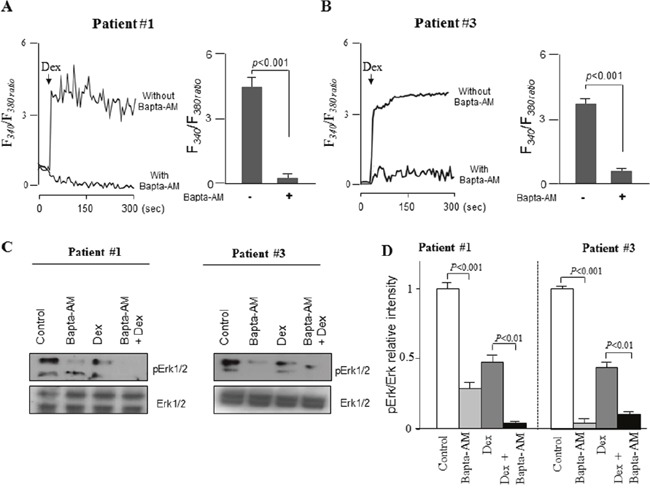
Bapta-AM potentiates dexamethasone-induced inhibition of ERK1/2 signaling by chelating Ca2+ signaling in primary blasts from ALL patients **(A, B)** Primary blasts were exposed to 100 nM dexamethasone (Dex) with or without preincubated (20 minutes) with Bapta-AM (5 μM) in Ca^2+^ buffer and the changes in [Ca^2+^]i were monitored as Figure 5. **(C)** ALL blasts were preincubated (20 min) with Bapta-AM (5 μM) in Ca^2+^ buffer and then exposed to 100 nM Dex for 5 min. Cell lysates were subjected to western blotting analysis with the indicated antibodies, along with quantification **(D)**. Data represent the mean ± SEM (n=3).

**Figure 9:**
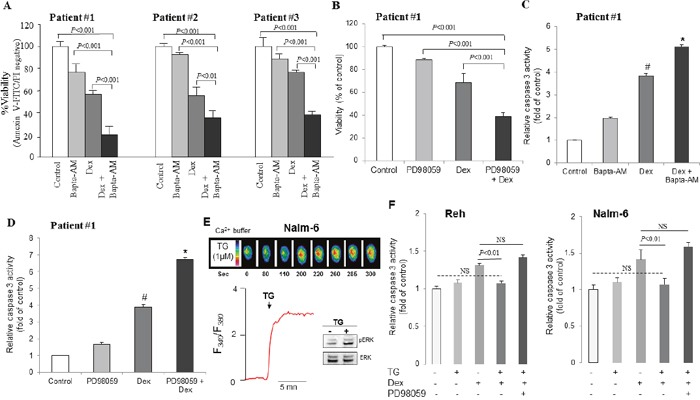
Dexamethasone-induced apoptosis is enhanced by chelating Ca2+ signaling and inhibition of ERK1/2 pathway in primary blasts from ALL patients **(A)** Apoptotic cells were measured by annexin V/FITC-PI staining followed by FACS analysis after 48 hours of treatment in ALL blasts. The percentage of cell viability was calculated by annexin V-FITC negative and PI-negative population and the values of control cells were considered as 100%. **(B)** Cell viability was detected by MTT metabolic colorimetric assay. The values of control cells were considered as 100%. Data represent the means ±SEM of triplicates. **(C, D)** Caspase 3 activity was measured with SAFAS Xenius XC Spectrofluorometer. *P<0.01 vs Dex alone treatment; #P<0.05 vs control. Data are mean ± SEM (n = 8). **(E)** Effect of thapsigargin (TG, 1 μM) on the changes in intracellular Ca^2+^ and ERK1/2 phosphorylation. **(F)** Effect of TG (100 nM) on Dex-induced caspase 3 activity. Data are mean ± SEM (n = 3).

To confirm the clinical relevance of these results, ALL blasts obtained from the peripheral blood of three leukemic patients were isolated and treated *ex vivo* with dexamethasone and/or Bapta-AM and PD98059. The results obtained correlate perfectly with those obtained in ALL cell lines. We observed that addition of dexamethasone (100 nM) induced a rise in [Ca^2+^]i in primary blasts from ALL patients (Figure 7A, 7B, 7C). dexamethasone-induced increases in [Ca^2+^]i were higher in Ca^2+^-containing, as compared with Ca^2+^-free, buffer (Figure 7A, 7B, 7C), suggesting, that dexamethasone significantly raised the peak of the Ca^2+^ elevation resulting from extracellular Ca^2+^ influx. In agreement, we observed that dexamethasone significantly boosted the subsequently evoked SOCE in primary ALL cells, which was blocked in the presence of SOCE inhibitor (Figure 7D). We also found that in the presence of Bapta-AM, dexamethasone failed to trigger cytosolic calcium elevation in blasts from ALL patient #1 (Figure 8A) and patient #3 (Figure 8B), as well as in patient #2 (data not shown). It was also found that dexamethasone slightly decreased the phosphorylation of ERK, whereas Bapta-AM completely suppressed ERK1/2 activation, suggesting that Ca^2+^ is a critical upstream factor that determined ERK1/2 phosphorylation (Figure 8C, 8D). Concurrently, in comparison to dexamethasone alone treatment, Bapta-AM greatly enhanced dexamethasone-induced inhibition of ERK1/2 phosphorylation, which may be due to the inhibitory action of this Ca^2+^ chelator on dexamethasone-induced Ca^2+^ influx (Figure 8C, 8D). Next, we assessed whether Bapta-AM or PD98059 would enhance primary ALL cells sensitivity to dexamethasone. Using apoptosis and MTT assays, we observed that cell viability of ALL patients was significantly decreased when cells were co-treated with dexamethasone (100 nM) and Bapta-AM (1 μM) or PD98059 (5 μM) compared with untreated cells or with cells exposed to these agents separately at the same doses (Figure 9A, 9B), confirming our above results observed in ALL cell lines. We next determined whether the influence of Bapta-AM or PD98059 on dexamethasone-induced apoptosis is associated with the activity of caspase-3. As shown in Figure 9, caspase-3 activity induced by dexamethasone was markedly potentiated by both Bapta-AM (Figure 9C) and PD98059 (Figure 9D). In addition, we next tested whether glucocorticoid-independent increases in [Ca^2+^]i levels could inhibit or protect ALL cells from dexamethasone-mediated cell death. Thus, the ability of thapsigargin (TG) to protect ALL cells from dexamethasone-evoked cell apoptosis was evaluated through the regulation of caspase-3 activity. TG induces a sustained Ca^2+^ influx in immune cells by depleting intracellular Ca^2+^ stores and stimulated ERK1/2 activation in a Ca^2+^-dependent manner [21]. In this study, we confirmed the effect of TG on cytosolic Ca^2+^ influx and observed that TG stimulated ERK1/ERK2 phosphorylation at the same time (Figure 9E), suggesting the implication of calcium influx in ERK activation, as demonstrated elsewhere [21]. Interestingly, pre-incubation with TG prevented dexamethasone-induced ALL cells apoptosis measuring by caspase-3 activation (Figure 9F). This inhibitory effect of TG on dexamethasone-stimulated caspase 3 activation may be due to the activation action of TG on ERK signaling pathway as prior addition of PD98059 prevented TG effect to curtail the dexamethasone-evoked caspase-3 activation in these cells (Figure 9F). These data together suggest that intracellular Ca^2+^-related ERK1/2 signaling pathway attenuates dexamethasone sensitivity by limiting caspase-dependent apoptotic pathway.

## DISCUSSION

Several studies have reported that disturbances in cellular calcium homeostasis are responsible for several diseases such as human lymphocytic leukemia [22]. Dexamethasone, a synthetic glucocorticoid, has been shown to increase or disrupt intracellular calcium homeostasis [7–10]. But, in most studies, glucocorticoids increased cytosolic calcium concentrations [23], and this is consistent with our observations reported here. In this study, we showed that dexamethasone induced a rapid increase in [Ca^2+^]i that was significantly reduced in Ca^2+^-free buffer in ALL cell lines and primary blast from ALL patients, suggesting that dexamethasone stimulates Ca^2+^ from intracellular pool, possibly via phospholipase C (PLC). Indeed, a selective inhibitor of PLC (U73122), curtailed dexamethasone-evoked Ca^2+^ signaling. As reported in cultured L6 myotubes [23], the present study confirms the participation of SOC channels in dexamethasone-mediated intracellular Ca^2+^ elevation. However, how this elevated-calcium stimulates dexamethasone sensitivity or resistance is not clear. Here, we provide evidence that pretreatment with intracellular Ca^2+^chelator (Bapta-AM) significantly prevented dexamethasone-induced elevation of [Ca^2+^]i level and potentiated dexamethasone-mediated apoptosis in ALL cell lines and primary blasts. ERK1/2 signaling pathway is a significant downstream substrate for Ca^2+^ ions, thus, increases in [Ca^2+^]i phosphorylated ERK1/2 proteins [13, 14]. Thus, we hypothesized that intracellular Ca^2+^ attenuates dexamethasone sensitivity and apoptosis by sustaining Ca^2+^-dependent ERK1/2 phosphorylation. We found that dexamethasone slightly decreased ERK1/2 phosphorylation in ALL cell lines and primary blasts, and depleting intracellular Ca^2+^ by Bapta-AM markedly enhanced inhibition of ERK1/2 pathway, suggesting a Ca^2+^-dependent mechanism. To shed light on the role of ERK1/2 activities on dexamethasone sensitivity, PD98059, a pharmacological inhibitor of ERK1/2 pathway [1], was used. We observed that PD98059 significantly enhanced dexamethasone-induced cell death. Our findings strongly suggest that Ca^2+^-related ERK1/2 activation attenuates dexamethasone sensitivity. Importantly, genomic data with functional screen have demonstrated that down regulation of mitogen-activated protein kinases (MAPKs) including ERK1/2, by shRNA increased glucocorticoid (prednisolone) sensitivity in ALL cells and primary blasts [2].

Mitochondria play a crucial role in the induction of cell apoptosis. It has been demonstrated that dexamethasone induced Δψ_m_ collapse and ROS production [7, 24]. In the present study, we found that intracellular calcium and ERK1/2 signaling pathway attenuate dexamethasone-induced Δψ_m_ collapse and ROS production in ALL cells. This is supported by the findings that chelating intracellular calcium with Bapta-AM or inhibiting ERK1/2 with PD98059 markedly enhanced dexamethasone-mediated Δψ_m_ collapse and ROS overproduction. These two events lead to mitochondria-mediated pro-apoptotic factors release such as cytochrome c and activity of caspase-3 [25]. In this report, we also noticed that by depleting intracellular calcium with Bapta-AM or inhibiting ERK1/2, the cytochrome c release and caspase-3 activation were significantly potentiated after dexamethasone exposure. These observations were supported in part by Rambal *et al*. [1], who have shown that MEK inhibitors potentiate dexamethasone-evoked cytochrome c release in ALL cells.

A pivotal question examined in this study is how intracellular calcium elevation by dexamethasone plays a central role in mediating dexamethasone sensitivity and resistance. It has been proposed previously that cytosolic calcium elevation by glucocorticoid is an important step in dexamethasone-evoked apoptosis, based on evidence that EGTA or Bapta reduced calcium signaling and protected CEM-C7 cells from glucocorticoid-induced apoptosis [26]. This is not supported by the findings reported in the current study. This difference might be due to the different cell types used in our current report and this earlier study, another possibility is due to the difference in calcium chelator concentrations used. Our finding is consistent with studies conducted using the same cells (i.e. CEM-C7) that shown that calcium chelator EGTA increased DNA fragmentation and enhanced dexamethasone effect, and concluded that massive calcium influx is not responsible for the initiation of glucocorticoid-mediated cell apoptosis [27]. In addition, studies conducted using mouse T-cells and DT40 cells that found that inhibiting IP3-gated calcium channels did not inhibit dexamethasone-induced apoptosis [28, 9]. Furthermore, previous findings demonstrated that glucocorticoid-induced bone cell death was prevented by the calcium-binding protein calbindin-D28k through ERK1/2 signaling pathway activation associated with caspase-3 inhibition [29].

Overall, our results suggest that intracellular Ca^2+^-related ERK1/2 signaling pathway attenuates dexamethasone sensitivity and apoptosis in ALL cells by decreasing Δψm collapse, ROS production, cytochrome c release from mitochondria, and caspase-3 activation. This report provides a novel resistance pathway underlying the regulatory effect of dexamethasone on ALL cells.

## MATERIALS AND METHODS

### Cell lines and reagents

The human leukemia cell lines Nalm-6 and Reh (DSMZ®, Deutschland) were cultured in RPMI 1640 medium containing 10% fetal bovine serum, 2 mM of L-glutamine with 5000 UI/L penicillin and 50 mg/L streptomycin (Eurobio®, France), and maintained at 37°C in a 5% CO_2_ humidified atmosphere. Fura-2/AM, Rhod-2/AM, Bapta-AM, U73122, SKF96365 and thapsigargin were purchased from Abcam Biochemicals, France. Poly-D-lysine, dimethylsulfoxide (DMSO), 2APB and dexamethasone were purchased from Sigma Aldrich, France. PD98059 was purchased from Euromedex, France. Antibodies to total (Erk1/2) and phosphorylated-Erk1/2 (pErk1/2) were obtained from Cell signaling Inc, France.

### Human tumor samples

Primary blasts from 3 patients diagnosed with ALL samples (peripheral blood) were used (Table 1). Informed consent was obtained following institutional guidelines and protocols were approved by the institutional review board of Rouen University and Hospital Center according to the Declaration of Helsinki. Mononuclear cells were isolated by Ficoll-hypaque centrifugation. Cells (> 80% blasts) were cryopreserved and stored in liquid nitrogen until use. After thawing, cells were maintained in RPMI 1640 medium supplemented as described above.

**Table 1:** Clinical and immunological characteristics on patients with ALL included in the study

Characteristics	Patient #1	Patient #2	Patient #3
Sex	F	F	F
Age at diagnosis (months)	20	104	1.5
Le (x 10^9^/L)	72	315	430
% of leukemic blasts	>80	> 85	> 90
Immunological phenotype	B	T	B
Cell surface molecules	CD10+ CD19+ CD22+ CD24+ CD38+ CD79a+	CD3+ CD5+ CD7+ CD10+ CD34+ CD38+	CD10- CD19+ CD22+ CD34+ CD36+ CD79a+
Cytogenetic	normal	normal	MLL/AF4+; t (4;11)+
MRD at day 35	-	< 0.5%	-
Chemotherapy(sensitivity)	+	+	-

Le: leukocytes numbers; ALL: acute lymphoblastic leukemia; MRD: minimal residual disease.

### Cell viability assay

Cultured cells (5 x 10^4^ cells/well) were seeded in 96-well culture plates with or without test compounds. After 48 hours, MTT assay was used to assess cell viability. Culture medium was aspirated, 100 μl serum free medium and 10 μl MTT solution (5 mg/ml) were added to each well, and the plates were incubated at 37°C for 3 hours. The medium was aspirated and 50 μl DMSO was added to each well to dissolve the formazan crystals. After 15 min at 37°C, the absorbance was read at 540 nm.

### Imaging and measurement of intracellular calcium [Ca^2+^]i levels

The cells were cultured on krystal 24-well glass bottom (Proteigene, France) coated with poly-D-lysine and then incubated with Fura-2/AM (5 μM) diluted in culture medium for 60 min at 37°C. After loading, cells were washed three times (600g x 10 min) and remained suspended in a buffer solution containing: 110 mM, NaCl; 5.4 mM, KCl; 25 mM, NaHCO_3_; 0.8 mM, MgCl_2_; 0.4 mM, KH_2_PO_4_; 20 mM, Hepes; 0.33 mM, Na_2_HPO_4_; 1.2 mM, CaCl_2_, pH adjusted to 7.4. For experiments in Ca^2+^-free medium, CaCl_2_ was replaced by EGTA (2 mM). SOCE was determined in Ca^2+^-free medium and subsequent Ca^2+^ re-addition in the presence of thapsigargin (1 μM) [20]. The fluorescence intensity of Fura-2/AM was measured at Ex 340 and 380 nm and Em 510 nm under the Leica DMI6000 B inverted microscope (Leica, France). The changes in intracellular Ca^2+^ were calculated as ΔRatio (F340/F380). Results were averaged from the individual cells (20-30 cells in a single run) with at least three independent experiments.

### Measurement of mitochondrial Ca^2+^ response, [Ca^2+^]m

Mitochondrial calcium ([Ca^2+^]m) was measured by loading ALL cells with the cell permeable Ca^2+^-indicator rhod-2/AM (2 μM; dissolved in DMSO) in RPMI for 1 h at 37°C, and then washed three times in rhod-2-free medium. To determine [Ca^2+^]m, rhod-2 was excited at λex (552 nm), and fluorescence was recorded at λem (581 nm).

### Mitochondrial membrane potential (ΔΨ_m_) measurement

Mitochondrial membrane potential was measured using the cationic dye JC-10 (a fluorescent ΔΨ_m_ dye, Santa Cruz Inc., Germany). This dye is red in polarized mitochondria and green in depolarized mitochondria. ALL cell lines were incubated with Bapta-AM (5 μM) or PD98059 (5 μM) for 30 min then stimulated with dexamethasone (100 nM) for 24 hours before loaded with 20 μM of JC-10 for 30 min at 37°C. After washing, the fluorescence intensity for both J-aggregates and monomeric forms of JC-10 was measured using SAFAS Xenius XC Spectrofluorometer (MC 98000 Monaco) at Ex 485 nm/Em520 and 595 nm.

### Intracellular reactive oxygen species (ROS) measurement

ALL cell lines were treated with Bapta-AM (5 μM) or PD98059 (5 μM) for 30 min then stimulated with dexamethasone (100 nM) for 24 hours. Cells were then loaded with 20 μM of Dihydrorhodamine 123 (DHR 123, a fluorescent probe for the detection of reactive oxygen species, Santa Cruz Biotechnology Inc., Heidelberg, Germany) for 30 min at 37°C. Cells were washed twice with PBS buffer after dye loading and the fluorescence intensity of Rhodamine 123 was measured with Spectrofluorometer SAFAS Xenius XC at Ex 500 nm/Em536 and by absorbance spectroscopy at 500 nm.

### ELISA cytochrome c measurement

ALL cell lines were treated with Bapta-AM (5 μM) or PD98059 (5 μM) for 30 min then stimulated with dexamethasone (100 nM) for 24 hours. The measurement of cytochrome c release was evaluated using the enzyme linked immunosorbent assay (ELISA) kits (Abcam Biochemicals, France) according to manufacturer's instructions.

### Measurement of caspase 3 activity

Activity of caspase-3 was measured using Ac-DEVD-AFC substrate (Enzo Life Sciences (ELS) AG, Villeurbanne, France), according to the manufacturer's instructions. Briefly, ALL cell lines were treated with Bapta-AM (5 μM) or PD98059 (5 μM) for 30 min then stimulated with dexamethasone (100 nM) for 24 hours, and then collected and lysed in cell lysis buffer (HEPES 50 mM, NaCl 100 mM, DTT 10 mM, CHAPS 0.1%, EDTA 1 mM, pH 7.4). 20 μg of cell lysate was added to 100 μL of caspase-3 buffer containing 40 μM of the caspase-3 substrate Ac-DEVD-AFC (fluorogenic) as final concentration and incubated at 37°C for 1 h in the dark. Caspase-3 activity was assessed by measuring fluorescence at excitation wavelength of 400 nm and emission wavelength of 505 nm using SAFAS Xenius XC Spectrofluorometer (MC 98000 Monaco).

### Apoptosis assay

Apoptotic cells were determined by Annexin V-FITC/PI staining (Abcam Biochemicals, France) according to the instruction. The percentage of annexin V-FITC and propidium iodide-positive cells were assessed by flow cytometry using BD FACSCanto II flow cytometer (BD Biosciences, USA). All flow cytometry data were analyzed using FlowJo 10.1 for Macintosh (Ashland, OR, USA).

### Cell cycle analysis

ALL cells were treated with or without test compounds for 48 hours and then washed with cold PBS and fixed with cold 70% ethanol for 30 mn at room temperature. After washing with PBS, cells were incubated in the dark for 15 mn with 1 mg/ml RNase A, 1 mg/ml propidium iodide. Cell-cycle distribution was determined by PI staining using flow cytometry and analyzed by FlowJo 10.1.

### Western blotting

ALL cell lines and primary blasts from ALL patients were incubated with Bapta-AM (5 μM) and PD98059 (5 μM) for 20 min and then stimulated with dexamethasone (100 nM) for 5 min, and lysed in 50 μl of RIPA buffer (Sigma-Aldrich, St-Quentin Fallavier, France) containing freshly added protease and phosphatase inhibitors (Pierce Biotechnology, Perbio Science, Rockford, USA). Proteins (40 μg) were loaded on 10 % SDS-PAGE gels and transferred to a nitrocellulose membrane. 5 % fat free milk in TBS with 0.1 % tween-20 (TBST) was used for blocking at room temperature for 1 h, then, membranes were incubated with anti-pErk1/2 and anti-Erk1/2 (Cell signaling, Ozyme, Yvelines, France) overnight at 4°C. After three times washing with TBST, blots were incubated with secondary antibodies-linked horseradish peroxidase (anti-mouse and anti-rabbit, Cell signaling, Ozyme, Yvelines, France) for 1 h at room temperature. Proteins were visualized with enhanced chemiluminescence reagents (GE Healthcare, Amersham, UK).

### Statistical analysis

Data are expressed as mean ± SEM values from at least three independent experiments. Statistical analyses were conducted with the PRISM Software (GraphPad Software, USA) using *t* test. Differences were considered statistically significant at p<0.05.
